# SARS-CoV-2 infected children form early immune memory responses dominated by nucleocapsid-specific CD8+ T cells and antibodies

**DOI:** 10.3389/fimmu.2022.1033364

**Published:** 2022-11-02

**Authors:** Karina Lima, Julia C. Fontoura, Priscila Oliveira de Souza, Tiago Fazolo, Gabriel Hilario, Renata Zorzetto, Luiz C Rodrigues Junior, Lais D. Coimbra, Alexandre Borin, Karina Bispo-dos-Santos, Fabiana Granja, Rafael Elias Marques, Gabriela Oliveira Zavaglia, Ingrid Rodrigues Fernandes, Fernanda Hammes Varela, Marcia Polese-Bonatto, Maiko Luís Tonini, Greice Madeleine Ikeda do Carmo, Walquiria Aparecida Ferreira de Almeida, Thiago J. Borges, Helder I. Nakaya, José Luiz Proenca-Modena, Sidia Maria Callegari-Jacques, Marcelo Comerlato Scotta, Renato T. Stein, Cristina Bonorino

**Affiliations:** ^1^ Departamento de Ciências Básicas da Saúde, Universidade Federal de Ciências da Saúde de Porto Alegre – UFCSPA, Porto Alegre, Brazil; ^2^ Brazilian Biosciences National Laboratory, Brazilian Center for Research in Energy and Materials (CNPEM), Campinas, Brazil; ^3^ Laboratory of Emerging Viruses (LEVE), Department of Genetics, Microbiology and Immunology, Institute of Biology, University of Campinas (Unicamp), Campinas, Brazil; ^4^ Biodiversity Research Centre, Federal University of Roraima (UFRR), Boa Vista, Brazil; ^5^ Social Responsibility – Programa de Apoio ao Desenvolvimento Institucional do Sistema Único de Saúde (PROADI-SUS ), Hospital Moinhos de Vento, Porto Alegre, Brazil; ^6^ Coordenação-Geral de Vigilância das Doenças de Transmissão Respiratória de Condições Crônicas, Departamento de Doenças de Condições Crônicas e IST, Secretaria de Vigilância em Saúde – Ministério da Saúde (CGDR/DCCI/SVS/MS)., Brasília, Brazil; ^7^ Departamento de Imunizações e doenças transmissíveis, Secretaria de Vigilância em Saúde - Ministério da Saúde (DEIDT/SVS/MS), Brasília, Brazil; ^8^ Center for Transplantation Sciences, Department of Surgery, Massachusetts General Hospital, Harvard Medical School, Boston, MA, United States; ^9^ Computational System Biology Laboratory (CSBL), Hospital Israelita Albert Einstein, São Paulo, Brazil; ^10^ Hub of Global Health (HGH), University of Campinas (Unicamp), Campinas, Brazil; ^11^ Departamento de Estatística, Universidade Federal do Rio Grande do Sul, Porto Alegre, Brazil; ^12^ Escola de Medicina, Pontifícia Universidade Católica do Rio Grande do Sul – PUCRS, Porto Alegre, Brazil; ^13^ Department of Surgery, University of California at San Diego – UCSD, La Jolla, CA, United States

**Keywords:** Memory T cell, antibodies, neutralizing antibodies, variants of concern, N protein, COVID-19

## Abstract

This is the third year of the SARS-CoV-2 pandemic, and yet most children remain unvaccinated. COVID-19 in children manifests as mostly mild or asymptomatic, however high viral titers and strong cellular and humoral responses are observed upon acute infection. It is still unclear how long these responses persist, and if they can protect from re-infection and/or disease severity. Here, we analyzed immune memory responses in a cohort of children and adults with COVID-19. Important differences between children and adults are evident in kinetics and profile of memory responses. Children develop early N-specific cytotoxic T cell responses, that rapidly expand and dominate their immune memory to the virus. Children’s anti-N, but not anti-S, antibody titers increase over time. Neutralization titers correlate with N-specific antibodies and CD8^+^T cells. However, antibodies generated by infection do not efficiently cross-neutralize variants Gamma or Delta. Our results indicate that mechanisms that protect from disease severity are possibly different from those that protect from reinfection, bringing novel insights for pediatric vaccine design. They also underline the importance of vaccination in children, who remain at risk for COVID-19 despite having been previously infected.

## Introduction

After almost three years of the SARS-CoV-2 pandemic, most children remain yet to be vaccinated against COVID-19. Children are often asymptomatic or develop mild disease upon infection. Some have questioned the need or urgency to vaccinate those under 18 years old, arguing that immunity to infection is sufficient for protection against the virus. Nevertheless, a recent study from the Center for Disease Control (CDC) (1) reports that the greatest increase in seroprevalence in the United States during 2022 was in children: 44.2 to 75.2% (0-11 years old) and 45.6 to 74.2% (12-18 years old). A recent US study reported an incidence of 316 cases of Multisystem inflammatory syndrome (MIS-C) per million of infected children (2). In Brazil, in 2022, until epidemiological week 24, 11,453 children (0-19 years old) were hospitalized for covid-19 and of these, 538 died. These epidemiological data are relevant and show the impact of SARS-CoV-2 infection for children not only for risk of COVID-19 disease, but also for possible and still unknown effects of SARS-CoV-2 infection (3). Some studies provide evidence that children may develop long COVID-19 (4, 5), reinforcing the need for a more thorough analysis of children’s immunity to infection by SARS-CoV-2.

Most of the information available on immune memory to the virus, whether generated by infection or vaccination, comes from studies on adults. Also, most of these studies focus on the responses against the Spike protein (S) of the virus, especially against its receptor binding domain (RBD). These currently constitute the source of data used to estimate the protective potential of immune responses. While different studies highlight the important role of innate immunity, especially of type I interferon (6), mechanisms and relative contributions of specific adaptive responses are less clear. Some propose neutralizing antibodies (NAbs) to the RBD as the best candidate correlate of protection (7) and others have shown that even when neutralization capacity wanes, T cells can still cross-recognize variants and protect against different SARS-CoV-2 variants (8), mostly because of T cell epitope conservation (9), with many of these epitopes come from sequences outside the S-protein coding region (10).

Fewer studies have focused on immune responses in COVID-19-affected children. We have previously compared immune responses in a sample of children and adults infected by SARS-CoV-2 (11), which revealed differences that might be related to protection from disease. The main difference observed was a predominant CD8^+^TNF-α^+^ T cell response against the nucleocapsid (N) protein in children. Here, we performed a longitudinal follow-up analysis of immune memory responses 3 and 6 months after infection. Children developed an early and sustainable CD8^+^TNF-α^+^ memory response to the N protein that is preferentially differentiated into terminal memory cells. This response is positively correlated with anti N, but not anti-S, antibody titers. Children produce durable antibody responses, however the neutralizing antibodies generated by infection do not efficiently cross neutralize other SARS-CoV-2 Variants of Concern (VOCs), such as Gamma and Delta lineages. Our results underline the need for a functional characterization of which responses generated by infection can prevent disease. They also reinforce the need for vaccination against COVID-19 in children, who even after infection remain at risk against new emerging SARS-CoV-2 variants.

## Material and methods

### Ethics statement

This study was approved by the Institutional Review Board (IRB 30749720.4.1001.5330) at Hospital Moinhos de Vento and Research Ethics Committee from Fundação Faculdade Federal de Ciências Médicas de Porto Alegre (CEP-UFCSPA) (CAAE 30749720.4.3001.5345). Legal consent was obtained from all participants or their legal guardians. The study was conducted according to good laboratory practices and following the Declaration of Helsinki.

### Patients

A prospective cohort study was carried out at Hospital Moinhos de Vento and at Hospital Restinga e Extremo Sul, both in Porto Alegre, southern Brazil, and their immune status at primary infection was assessed as described (11). Study participants, adults and children older than 7 months, were asked to return for a 3- and 6-month follow-up visit to the respective hospital between October 2020 and April 2021 for blood sample collection.

### PBMC isolation and *in vitro* T cells stimulation assays

Blood was collected in EDTA tubes (Firstlab, PR, Brazil) and stored at room temperature before processing for PBMC isolation and plasma collection. Plasma was separated by centrifugation and cryopreserved. PBMCs were next isolated by density-gradient centrifugation using Ficoll–Paque™ PLUS (GE Healthcare^®^), and either studied directly or resuspended in fetal bovine serum (FBS) 5% DMSO and stored in liquid nitrogen until use. For *in vitro* T cell stimulation, cells were thawed, assayed for viability, counted, and plated in 96-well plates at 3×10^5^ PBMCs/mL, at 100 µL/well in RPMI1640 medium (Sigma-Aldrich - R8758) supplemented with 10% FBS and 100 IU of penicillin/mL, 100 μg of streptomycin/mL (Lonza, Belgium) and 2 mM L-glutamine (Lonza, Belgium) (R10H medium). Subsequently, cells were stimulated with peptide PepTivator SARS-CoV-2 Prot S (130-126-700 - Miltenyi Biotec, Germany), PepTivator SARS-CoV-2 Prot N (130-126-698 - Miltenyi Biotec, Germany) and PepTivator SARS-CoV-2 Prot M (130-126-702 - Miltenyi Biotec, Germany) at 1 μg/mL. Anti-CD3/anti-CD28 (4 μg/mL/2 μg/mL, BD Biosciences) were used as a positive control for stimulation and used to normalize values obtained by peptide stimulation. All treatments were incubated for 18h at 37°C and 5% CO_2_. Three hours before harvesting, Golgi Plug (BD Biosciences, USA) 1 μg/mL was added to each well. Cells were stained and phenotypes were analyzed by flow cytometry.

### Flow cytometry analysis

After *in vitro* stimulation, cells were stained with BD Horizon™ Fixable Viability Stain 510 together with antibodies for surface markers, as follows: anti-CD4-PerCP-Cy5.5 (clone RPA-T4), anti-CD8-APC-H7 (clone SK1), anti-CCR7-BV421 (clone 2-L1-A), CD45RA-PE-Cy7 (clone L48). They were subsequently fixed and permeabilized using Cytofix/Cytoperm kit (BD Biosciences-Pharmingen, USA), then stained with anti-IFNγ-FITC (clone 4S.B3), anti-TNF (clone MAb11) and anti-IL-17-PE (clone SCPL1362) antibodies. Antibody dilutions are available upon request. All samples were analyzed using BD Biosciences - FACSCanto II and FlowJo 10.7.1 software.

### Enzyme-linked immunosorbent assay

Plasma was tested for IgG and IgA antibodies to S-RBD protein (#RP-87678 - Invitrogen, USA), trimeric SARS-CoV-2 spike (S), S protein from variants Delta and Gamma (kindly provided by Dr. Leda Castilho - Federal University of Rio de Janeiro - UFRJ, Brazil) and N protein (kindly provided by Dr. Ricardo Gazinelli - Fiocruz Belo Horizonte, Brazil) using a protocol described in ^17^. Briefly, ELISA plates (Kasvi, Brazil) were coated overnight with 1 μg/mL of SARS-CoV-2 proteins. On the following day, plates were blocked for 1 h at room temperature with a blocking buffer (3% skim milk powder in phosphate-buffered saline (PBS) containing 0.05% Tween-20). Plasma samples were heat-inactivated at 56°C for 60 minutes and then serially diluted in 1% milk in 0.05% PBS-Tween 20 starting at 1:25. Plasma was incubated for 2 h at 37°C. Secondary antibodies were diluted in 0.05% PBS-Tween and incubated for 1 h at room temperature. For both IgG, anti-human peroxidase produced in rabbit (#IC-1H01 - Rhea Biotec, Brazil), and IgA, anti-human peroxidase produced in goat (#A18781 - Invitrogen, USA), was used at a 1:10,000 dilution. TMB Elisa Substrate - High Sensitivity (Abcam, United Kingdom) was added for 30 minutes, and the reaction stopped with 1M chloric acid. Results were documented in an ELISA reader (Biochrom EZ 400), and O.D. at 450 nm was used to calculate the area under the curve (AUC), using a baseline of 0.07 for peak calculation ^18^. For average AUC calculations, adult individuals who were vaccinated between the 3 and 6-months timepoint were excluded.

### Viruses and cells

The present study was conducted using three different SARS-CoV-2 lineages: The B original lineage (Genbank HIAE-02 SARS-CoV-2/SP02/human/2020/BRA) isolated from the second case of COVID-19 in Brazil and kindly provided by Prof. Edison Durigon; Gamma (P.1) (GISAID: EPI_ISL_1708318) isolated from a patient seen in Campinas, Brazil; and Delta (B.1.617.2-like) (GISAID: EPI_ISL_3461104) isolated from a patient seen in Porto Alegre, Brazil. SARS-CoV-2 lineages were isolated and propagated in Vero cells (CCL-81; ATCC, Manassas, VA, USA) as described elsewhere (Clarke et al.,2022). Viral titers of the viral stocks used in the study were determined by plaque-forming unit (PFU) assay as previously described (12). All experiments with viable SARS-CoV-2 were performed under biosafety level 3 (BSL-3) in the Laboratory of Emerging Viruses (LEVE) of the University of Campinas, Brazil.

### Plaque reduction neutralization test

Patient’s serum neutralizing activity against B, Gamma and Delta isolates of SARS-CoV-2 were evaluated using an in-house Plaque Reduction Neutralization Test (PRNT) as previously described (13). Briefly, plasma samples from infected patients were serially diluted in a 1:2 dilution factor (1/10, 1/20, 1/40, 1/80, 1/160 and 1/320) in DMEM and incubated with a solution containing 2x10^3^ PFU/mL of SARS-CoV-2 isolates for 1h at 37° C with 5% CO_2_. Virus-serum mixtures were added to Vero cells monolayers and incubated for an additional hour for viral adsorption. Supernatants were then discarded, and Vero cells were covered with carboxymethylcellulose (CMC) 1% in DMEM containing 5% FBS. Cells were incubated for 72h to 96h at 37°C with 5% CO_2_. CMC/DMEM medium was removed, cells were fixed with 8% paraformaldehyde and stained with 1% methylene blue (Sigma-Aldrich). After staining, the number of lysis plaques in all wells were manually counted and the plaque reduction was calculated for each patient sample after normalization with the plaque numbers observed in SARS-CoV-2 positive controls, without serum. PRNT_50_ for each patient was calculated as the dilution that showed approximately a 50% reduction in the number of plaques when compared with the SARS-CoV-2 infected positive controls.

### Statistics

Patients´ demographic or clinical characteristics measured as categorical variables were described using percentages and compared among groups using Pearson chi-square test. Continuous clinical characteristics were summarized in terms of median and interquartile range (IQR) and compared among groups using Kruskal-Wallis test. Differences among groups of patients for time variation in levels (area under the curve, AUC) of IgG and IgA antibodies were analyzed using Generalized Estimating Equations (GEE). The models considered gamma distribution for the data, log link and unstructured working correlation matrix. Sequential Sidák’s adjustment was used to adjust p-values for pairwise multiple comparisons. These calculations were done using SPSS v.18. For memory T cell data, the results observed in the three time points were compared using nonparametric Friedman test in each patient´s group. If the result was statistically significant, Wilcoxon test for paired samples was then employed to compare 3- and 6-month. Memory T cell difference among groups in selected time points of collection were analyzed using Kruskal-Wallis nonparametric analysis of variance, followed by Dunn´s test for pairwise comparisons between groups. Spearman´s rank correlation matrices were obtained using R package corrplot (14).

### High-dimensional data and heatmap analysis of flow cytometry data

t-SNE and FlowSOM analyzes were performed on FlowJo v.10.8.1. Cells populations were downsampled from live cells, using the Downsample v.3.0.018 plugin for Flowjo to 2000 cells and subsequently concatenated. FlowSOM clustering with the FlowSOM v3.0.18 plugin was performed on the concatenated file with n= 6 clusters for the memory T cells populations. The FlowSOM output was surface markers expression and the percentage of cells in each cluster between groups. The t-SNE map was built with a vantage point tree algorithm with 550 iterations. Cluster identification in the map was performed with the help of the Cluster Explorer v1.6.3 plugin through the heatmap expression of surface markers CD4, CD8, CD45RA, and CCR7. Heatmap analyses were performed using the ggplot2 package v3.3.6 (15), where data was normalized through z-score, considering CD8, CD4, CD45RA, CCR7, TNF-α, IFN-γ and IL-17 markers.

## Results

**Figure 1:**
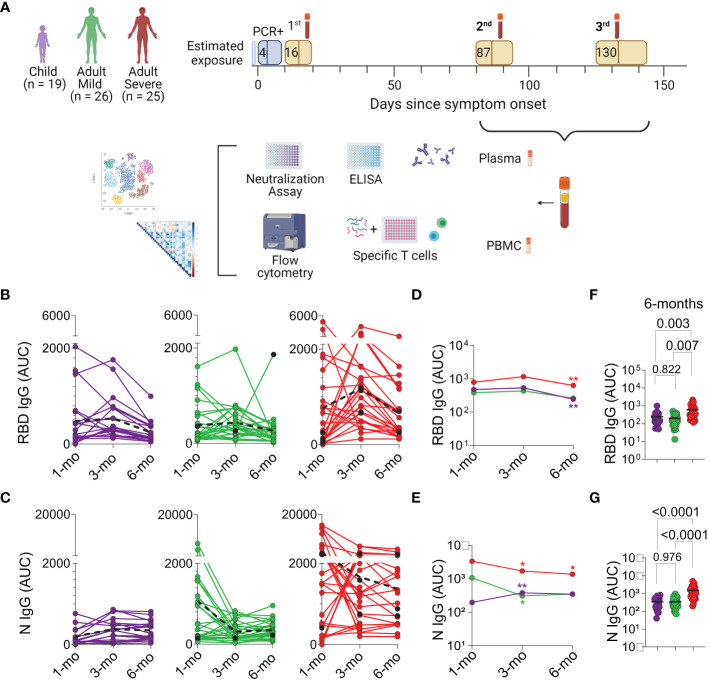
Study design and longitudinal Analysis of Antibodies. **(A)** Intervals of second and third blood draw from cohort patients, followed by cryopreservation and analysis. **(B, C)** Plasma IgG antibody binding to SARS-CoV-2 RBD and N protein, respectively, determined by enzyme-linked immunosorbent assay (ELISA), (n=70). Longitudinal analysis through connecting lines, color-coded, represented as area under the curve (AUC). Dotted lines in black highlight median values. Individuals colored in black were vaccinated between the second and the third collection point (Mild with Astrazeneca and Severe with Sinovac). **(D, E)** Median titers plotted over time. Differences among group profiles were analyzed using Generalized Estimating Equations (GEE) and sequential Sidák’s adjustment for pairwise multiple comparisons, colored asterisk (*) indicate statistical difference compared to previous point of collection. **(F, G)** Individual AUC 6 months after acute infection, in the three groups of patients; The horizontal bar is the median, P values are displayed over brackets. Graphical study design was created with biorender.com.

The study design is summarized in **Figure 1A**. The cohort included children, adults with mild and severe disease, with blood collected between July 2020 and April 2021. All children only had mild symptoms, as previously described in (11). Patients, or their legal guardians, consented to donate blood samples approximately 3 and 6 months after the first sample was collected at diagnosis (11). Samples were cryopreserved and later analyzed for memory markers including specific antibodies and T cell populations. Demographic and clinical data of patients that remained in the study for six months are presented in **Table 1**. The percentage of absence between the sample studied for acute infection (11) versus memory response study, was 21% in children, 8% in adults with mild diseases and 24% in adults with severe disease.

**Table 1:** Clinical characteristics of patients.

Characteristics	Child(N = 19)	Mild(N = 26)	Severe(N = 25)	P-value
Age (y), median (IQR)	12.0 (3.7–15.2)	42.5 (29.7–47.5)	51.0 (38.0–70.5)	**<0.001**
Female sex, n (%)	10 (52.6)	16 (61.5)	12 (48.0)	0.616
**Days from symptom onset to sample collection**				
1^st^ point, days, median (IQR)	16 (12.0–42.0)	18 (16.0–22.5)	11 (9.5–15.5)	0.063
2^nd^ point, days, median (IQR)	87 (83.0–106.0)	92 (84.0–100.0)	82 (74.5-88.0)	**0.019**
3^rd^ point, days, median (IQR)	131 (119.0–155.0)	133 (127.0–143.5)	125 (121.5-130.5)	0.080
Days from symptoms to SARS-CoV-2 positive PCR test, median (IQR)	3 (2–8)	3 (2–5)	7 (3.5–10.0)	**0.012**
Number of symptoms, median (IQR)	7 (4-11)	14 (10-19)	12 (8-17)	**0.003**
Number of underlying medical conditions, median (IQR)	0 (0-1)	0 (0-1)	2 (1-6)	**0.005**

Quantitative variables were compared using Kruskal-Wallis test and for categorical variables Pearson’s Chi-squared test was used; IQR, interquartile range. Bold values indicate P-value < 0.05

### IgG titers to the N, but not the S protein, increase over time in children

Antibodies from blood samples are currently the main surrogate markers of memory of infection and/or vaccination. Plasma titers of IgG antibody binding to SARS-CoV-2 RBD (**Figures 1B, D, F**) and N (**Figures 1C, E, G**) proteins, as well as of IgA (**Figure S1**) were determined by ELISA immediately after acute infection and at the 3 and 6 months after infection. These two later time points values were compared to the those detected at diagnosis. Considerable individual variation in responses to both antigens, a hallmark of infection, can be observed in each group (**Figures 1B, C**). Remarkably, children’s anti-N IgG titers show a significant increase (p=0.001) at the 3 months timepoint, and which is maintained until 6 months (**Figure 1E**), while their anti-S-IgG titers decrease significantly (p<0.001) (**Figure 1D**). Although adults with severe disease show significantly higher titers of both anti-N and anti-S antibodies throughout time, a significant decrease in both antibodies is observed at the 6 months post-acute infection (anti-S IgG p<0.001; anti-N IgG p=0.026) (**Figures 1D–G**).

IgA titers to the RBD dropped significantly in all groups at this time point, while anti-N IgA antibodies increase slightly in children, and significantly in adults which had mild disease (p<0.001) (**Figure S1C** and **S1D**). These results indicate that a test based solely on anti-IgG RBD might not constitute the best assay to detect antibody memory in most of the population, especially in children.

Vaccinated individuals are depicted in black (**Figures 1B, C**; and **Figures S1A, S1B**). Their titers are shown for comparison but were excluded from the average AUC calculations. One adult with mild disease was vaccinated and received the Astra Zeneca vaccine; the adults with severe disease that were vaccinated received the Sinovac vaccine. The latter showed no obvious increases in antibodies. Among individual children, which were all unvaccinated at that time, no obvious increases in antibody titers were detected at the analyzed timepoints. This is consistent with the time in which the study was carried out, that is, on lockdown and could indicate that these children were not reinfected during that six-month period.

### Memory neutralizing antibodies in children

**Figure 2:**
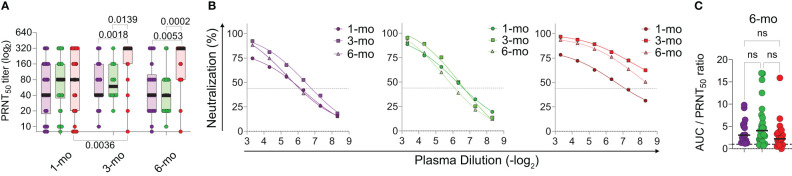
Longitudinal analysis of neutralizing antibody titers by PRNT. **(A)** Individual PRNT50 titers over time. **(B)** Comparison of mean neutralization curves at each collection point, per group. **(C)** Antibody potency analysis between groups through analysis of ratio between S RBD antibody/PRNT 50 antibody neutralization. Differences between patients’ groups at the same collection point and differences between collection points were assessed by Kruskall Wallis’ and Dunn’s *post hoc* test. ns, non-significant.

More than total specific antibody titers, the detection of neutralizing antibodies (NAbs) is frequently proposed as the gold standard for protective immunity to different viruses, including SARS-CoV-2. We thus performed a longitudinal analysis of NAb production in the cohort members (**Figure 2**). Once again, considerable individual variation can be observed. All three groups show higher averages in PRNT50 titers at the 3 months timepoint (**Figures 2A, B**) in comparison with the diagnosis timepoint, although this increase is significantly higher in adults who had severe disease (p<0.01). At the 6 months timepoint, NAbs titers are maintained, and still higher in the adults who experienced severe disease (**Figures 2A, B**). Finally, the ratio between total and neutralizing antibodies, at the 6 months timepoint (**Figure 2C**) was not different among the groups. Altogether, these results suggest that SARS-CoV-2 infected children make and sustain neutralizing antibodies at levels that are comparable those observed in adults.

### Dynamics and quality of specific T cell memory generation to SARS-CoV-2 are different in children compared to adults

The low antibody titers shown by children over time indicate that the mild form of disease they experience might be explained by other mechanisms, particularly those mediated by T cells. To investigate the generation of memory T cell responses in the cohorts, we stimulated PBMCs from 3 and 6 months after the acute infection with peptide pools of SARS-CoV-2 proteins S, M or N. SARS-CoV-2 specific T cells were then analyzed by flow cytometry for T cell memory (using CD45RA and CCR7 expression) and functional markers through the production of cytokines (IFN-γ, IL-17 and TNF-α).

**Figure 3:**
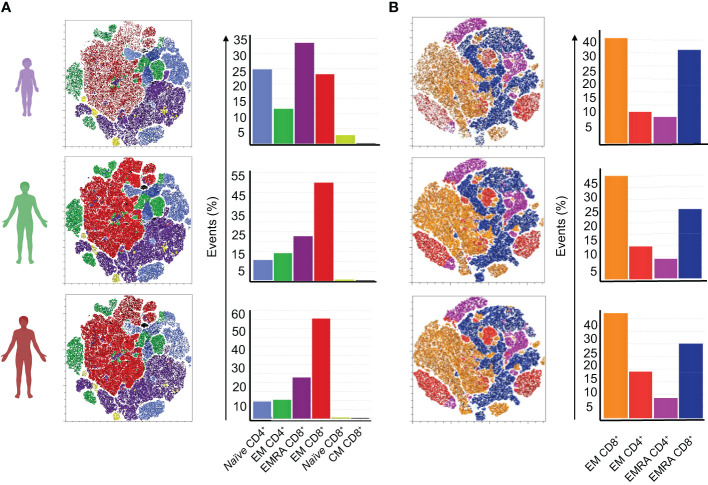
Generation dynamics of specific memory T cell responses at 2^nd^ and 3^rd^ collection points. An unbiased tSNE analysis was performed for analysis of memory T cells populations, stimulated with peptide pools of the S, N, and M proteins of SARS-CoV-2, gated from live cells. Clusters were characterized by the expression of surface markers CD4, CD8, CD45RA, and CCR7. **(A)** Clusters at the second time point, from a total of 36 samples (8 children, 17 mild, 11 severe); **(B)** Clusters at the third time point, from a total of 29 samples (7 children, 12 mild, 10 severe). Bar graphs show frequencies of events in each of the seven most frequent clusters identified in each time point. Color legends identify clusters as interpreted based on their surface markers.

An unbiased tSNE analysis of SARS-CoV-2 specific stimulated T cells (CD4 and CD8) was performed. Seven main clusters in the 3- and 6-months points were identified. At 3 months (**Figure 3A**), children show generally lower frequencies of specific T cells than adults. Both adult groups show a higher frequency of a cluster of CD45RA^-^CCR7^-^ CD8^+^ T cells – which we named effector CD8^+^ T cells (EM CD8^+^). Children presented fewer events for this cluster and more events for the clusters of CD45RA^+^CCR7^+^ CD4^+^ as well as CD45RA^+^CCR7^+^ CD8^+^, named by us *naïve* CD4^+^ and CD8^+^ T cells, respectively. Notably, one cluster of CD45RA^+^CCR7^-^ CD8^+^ cells, identified by us as terminally differentiated memory (EMRA) T cells that emerge at this point is most frequent in children compared to either of the adult groups. At 6 months, clusters were qualitatively different (**Figure 3B**). Frequencies of events in each of the identified clusters were more similar between children and adults. Children no longer present prominent clusters of *naïve* cells. Instead, CD8+ EMRA cells were dominant in children, while the cluster characterized by effector cells is more evident in adults. In sum, these results indicated that memory CD8^+^ T cell responses in children were faster, and more intense, than in adults.

**Figure 4:**
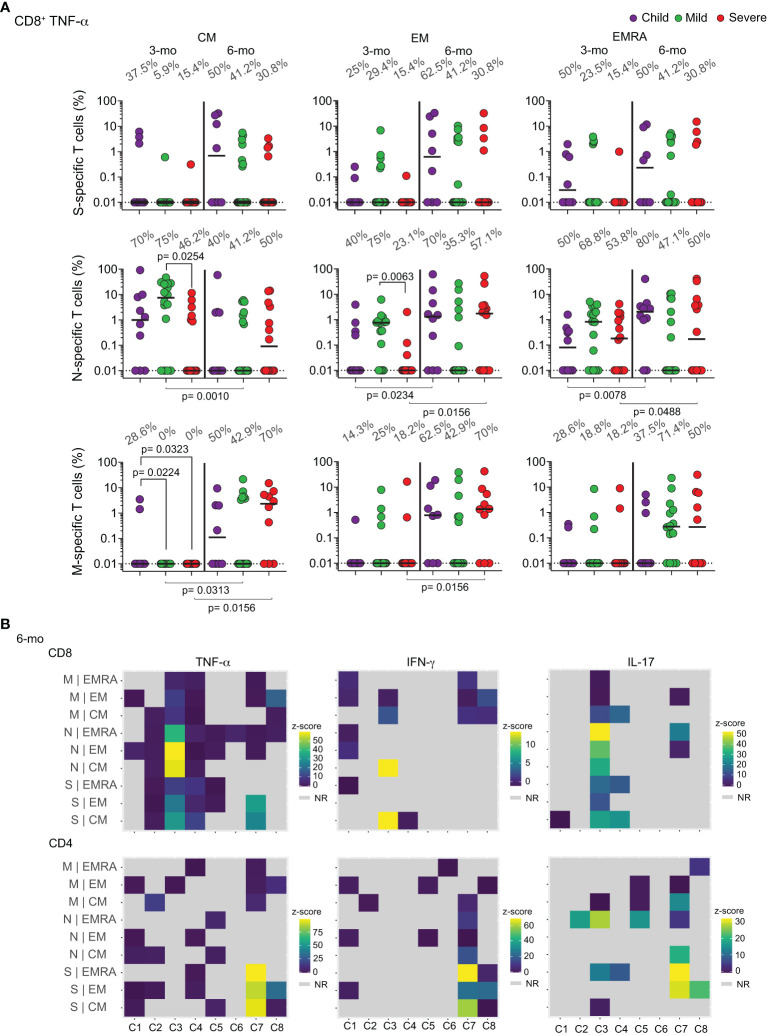
Longitudinal analysis of functional specific memory T cells. **(A)** Individual frequencies of TNF-α production in memory T CD8^+^ cells collected at the 3 and 6-months post-acute infection, responding to *in vitro* challenge with different SARS-CoV-2 peptide pools to proteins S, N, or M, percentages of responders are indicated above columns. Differences between patients’ groups at the same collection point were assessed by Kruskal-Wallis and Dunn’s *post hoc* test; differences between collection points were assessed by Wilcoxon test. **(B)** Heatmap illustrating CD8^+^ and CD4^+^ responses of 8 individual children to the different SARS-CoV-2 peptide pools. Data is shown as z-score, where patients who did not respond to stimuli are considered in light gray. C – individual children. NR – Non-responsive. CM – Central memory; EM – Effector memory; EMRA – Terminally differentiated effector memory cells.

We next proceeded to a more quantitative and functional analysis of these memory T cell responses. We had previously shown that anti-N CD8^+^TNF-α^+^ T cells were generated upon acute infection in children, while adults focused that response on S peptides (11). To investigate if this was the main response that was differentiated into memory in children, we performed a longitudinal analysis of the percentages of specific cells producing TNF-α, IL-17 or IFN-γ in response to the different peptide pools (**Figure S2**). The results are shown in **Figure 4** and **Supplementary Figures 3** and **4**. **Figure 4A** shows that after 3 months most children show central memory CD8^+^TNF-α^+^ T cells responding to N peptides. Some children already show effector memory and even EMRA cells with these characteristics. At the 6-month time point, most children had developed CD8^+^TNF-α^+^ effector memory and EMRA cells specific for N peptides, but not for the S and M peptide pools. This does not happen for IFN-γ and IL-17 CD8 memory responses (**Figures S3A, S3B**). While most children and adults generate memory cells producing these two cytokines in response to all three proteins at the 3 months post-acute infection, most of these responses are lost at 6 months. **Supplementary Figure 4** shows quantification of functional specific CD4^+^ T cell responses at the two timepoints. It is remarkable that most children lose at 6 months all the detectable circulating responses seen at 3 months post-acute infection. These results suggest that children, but not adults, develop early and long-lasting T cell memory responses, and that CD8^+^TNF-α^+^ cells specific for the N protein constitute a major, if not the main, component of those responses.

To further investigate which children are forming memory responses to the viral peptides, we compared their individual responses through heatmaps (**Figure 4B**). Most children focused their memory responses on CD8^+^TNF-α^+^, especially to the N protein, with fewer individuals targeting the M and S peptide pools, at 6 months after acute infection.

**Figure 5:**
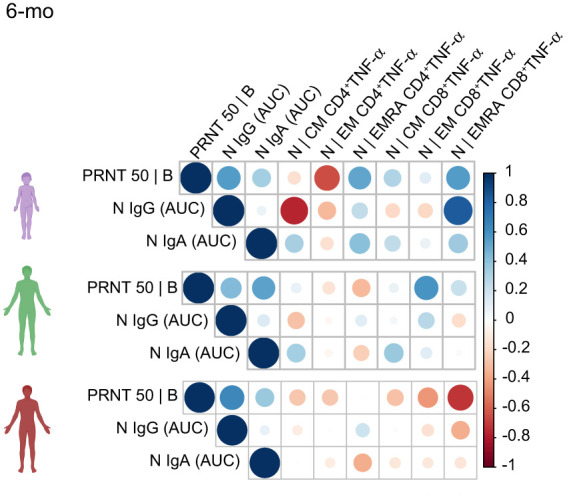
Spearman correlation analysis of antibody and N-protein specific TNF-α+ T cell responses 6-months after acute infection. The matrix represents the correlation analysis of N-specific effector T cells responses (in percentages of CD4^+^ and CD8^+^ cells expressing cytokine TNF-α in response to the peptide pools), neutralizing antibodies to the B original variant, as well as the antibody response to the N protein (represented as values for the AUC – area under the curve). Total of 32 samples (children = 9, mild= 16, severe = 10).

A Spearman correlation analysis (**Figure 5**) evidenced that in children, but not adults, anti-N IgG and IgA titers correlated positively with N-specific CD8^+^TNF-α^+^ T cells – especially EMRA cells. PRNT50 titers in children, but not in adults, also correlated positively with CD8^+^TNF-α^+^ EMRA cells. This positive correlation was not observed for protein S (**Figure S5**). Altogether, these results indicate that children generate distinctive memory T and B cell responses to SARS-CoV-2 infection, in kinetics, target and function.

### Memory NAbs in children do not efficiently cross-neutralize infection by emerging SARS-CoV-2 variants

**Figure 6:**
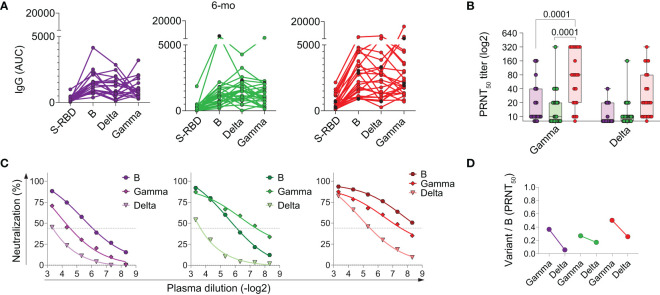
Impact of variant emergence on memory antibody recognition 6 months after acute infection. **(A)** Total individual IgG binding to the RBD compared to trimeric B and variants spike protein, by enzyme-linked immunosorbent assay (ELISA); **(B–D)** PRNT analysis and fold reduction of recognition of neutralizing antibodies. Differences between patients’ groups were assessed by Kruskal Wallis’ and Dunn’s *post hoc* test.

At the time we ended sample collections for this study, the original variant, B, was being substituted for more prevalent variants of concern (VOCs), namely Gamma (16), and later, Delta (17). The neutralizing antibodies generated by vaccination encoding the RBD of the S protein were shown to retain protection potential against VOCs (16). Most of the children, however, have not been vaccinated. We asked if the children’s memory antibody responses to SARS-CoV-2 infection would be protective against infection by the emerging variants. To investigate that, we analyzed the recognition and neutralizing potential of the memory antibodies present at 6 months post-diagnosis. We assayed these antibodies against the trimeric Spike of the variants by ELISA and performed PRNT tests using the Gamma and Delta lineages (**Figure 6**). Responses to the trimeric S of the original variant (B) were always higher in titers than responses to the RBD (**Figure 6A**). For almost every individual, total IgG antibodies to the B trimeric S are still able to recognize the trimeric S of variants Gamma and Delta. However, this does not hold for neutralizing antibodies (**Figures 6B–D**). Only adults with severe disease maintained significant higher potential for neutralization of the Gamma VOC compared to children and adults with mild disease. For Delta, a later-emerging VOC, marked reductions in neutralization are observed for all groups. These results indicate that neutralizing antibodies generated by infection with the original B variant in children have low or no protective potential upon encounter with emerging VOCs.

## Discussion

Throughout the SARS-CoV-2 pandemic, children have been regarded as having a minor risk of disease, mostly because of the mild presentation of COVID-19 observed in so many of them. They have thus become the least studied age group, and the last to be vaccinated. However, the immune responses they develop to infection might hold important information on what is necessary to generate fast and efficient protection against disease. In this study, we identified key differences in the memory immune response generated by infected children when compared to adults. The increase of anti-N antibodies three months after diagnosis and the early appearance of memory N-specific CD8^+^TNF-α^+^ cells could reflect a higher precursor frequency of N-peptide-recognizing cells in children. This could be due to pre-existing cross-reactive T cells and might explain the mild disease generally presented by SARS-CoV-2 infected children. The N gene has a higher degree of conservation among different lineages of SARS-CoV-2 than the S gene, accumulating fewer mutations (18). It is highly expressed during infection (19) and is associated with the protection of the viral RNA genome, resulting in specific T cells that are long-lasting (20). In adults, cross-reactive memory T cells were shown to be associated with protection from COVID-19 (21). In that study, the peptides recognized by cells in protected individuals were mainly from the nucleocapsid (N) protein. Most, but not all, individuals who recover from COVID-19 generate T cell responses to multiple viral epitopes (22) and a strong activation of these cross-reactive clones has been linked to protection (23). It has been suggested that, in children, a high frequency of antibodies and T cells recognizing antigens from seasonal coronaviruses could be due to repeated reinfections (24), though the protective immunity generated by infection with these endemic coronaviruses is not usually long-lasting (25). Studies on precursor frequencies of T cells recognizing coronaviruses epitopes, however, are lacking. This makes it difficult to support such a hypothesis with evidence. Interestingly, the efficacy of RBD-encoding mRNA vaccines drops from over 90% in adults to 51% (Moderna) and 75% (Pfizer) in young children (26). It is possible that a better tailoring of vaccines for children, including N peptides, could stimulate this N-specific response, thus increasing efficacy in this age bracket.

The fact that the N-specific antibodies in children, but not adults, correlated with anti-N CD8^+^TNF-α^+^ memory T cells may evidence functional mechanisms of protection that have not been characterized for COVID-19. While a correlation between antibodies and CD4^+^ T cells, mainly follicular T cells (Tfh), would be expected, a recent study by Nelson et al. (27) analyzed memory T cell responses using peptide:HLA tetramers and did not find high associations between peak antibodies to SARS-CoV-2 and Tfh cells. Klarquist et al. found that B cells are necessary to produce IL-27 that enhances CD8^+^ T cell responses (28). Thus, in the case of the children’s anti-N response, antibodies might be markers for a CD8+ T - B cell crosstalk that only recently started being characterized. Importantly, neutralization titers generated by infection were not sufficient to cross-neutralize emerging VOCs in a plaque reduction assay. These results concur with epidemiological reports of peak reinfections in children occurring during the Gamma and Delta variant waves (29, 30). They also underline differences in the protective potential of antibodies generated by vaccination, that can cross-neutralize variants, versus those generated by infection. Immune responses that control disease in children may thus be different from those that control infection. Studies that further investigate these differences are sorely needed. There have been, however, a few studies showing that children develop a strong S protein response. Chiara, et al. observed that younger children develop higher levels of binding antibodies, while Dowell et al, observed that children developed higher antibody responses to the spike protein when compared to adults. In addition, they observed that children developed N- and M-specific T cell responses lower than those of adults (31% children x 68% adults). These differences between data could be due to MHC variations between cohorts, differences in vaccine schedule and adherence, as well as to the seasonal coronaviruses in circulation during each study. One limitation of this study is the small number of participants. This study was conducted during 2020-2021, and some children were not brought back for all blood draws at the follow-up time points. Nevertheless, the consistency of responses found in all our analyses indicates important aspects of pediatric COVID-19 immunity, that may inform vaccination strategies for this age group (31, 32).

## Data availability statement

The data presented in the study are deposited in the Mendeley repository, found here: https://data.mendeley.com/datasets/pfkhyry6wt/1. Further inquiries can be directed to the corresponding author/s.

## Ethics statement

This study was reviewed and approved by Institutional Review Board (IRB 30749720.4.1001.5330) at Hospital Moinhos de Vento and Research Ethics Committee from Fundação Faculdade Federal de Ciências Médicas de Porto Alegre (CEP-UFCSPA) (CAAE 30749720.4.3001.5345). Written informed consent to participate in this study was provided by the participants’ legal guardian/next of kin.

## Author contributions

KL, JF, PS, TF, all designed and performed experiments, analyzed data, wrote, and edited the manuscript. KL, performed and analyzed t-distributed stochastic neighbor embedding (tSNE) analysis. JF, performed and analyzed the correlation and heatmap matrices. GH, RZ, assisted in experiments. LR, helped design the study. LC, AB, KB-d-S, FG, RM, performed Plaque Reduction Assay (PRNT). GZ, IF, FV, MP-B helped collect patients. MT, GC, WA, assisted with epidemiological information. TB helped design the study and edited the manuscript. HN helped with tSNE and heatmap analysis and edited the manuscript. JLPM, coordinated and performed PRNT analysis and edited the manuscript. SC-J coordinated all statistical analysis and edited the manuscript. MS, RS coordinated the cohort, conceived, and designed the study and edited the manuscript. CB, conceived and designed the study, performed data analysis, wrote, and edited the manuscript. All authors contributed to the article and approved the submitted version.

## Funding

Funding for this study was provided by PROADI - HMV, and the Ministry of Health; fellowships for KL, JF, RS and CB are from CNPq; fellowships for GH, PS and TF are from CAPES. TB is a recipient of an American Heart Association fellowship grant.

## Acknowledgments

We wish to thank Caroline Nespolo de David for assistance in project management and the COVIDa consortium (a full list of consortium members appears in Supplementary Note 1). Finally, we wish to thank all the patients who accepted to enroll in the study and donate blood.

## Conflict of interest

The authors declare that the research was conducted in the absence of any commercial or financial relationships that could be construed as a potential conflict of interest.

## Publisher’s note

All claims expressed in this article are solely those of the authors and do not necessarily represent those of their affiliated organizations, or those of the publisher, the editors and the reviewers. Any product that may be evaluated in this article, or claim that may be made by its manufacturer, is not guaranteed or endorsed by the publisher.
