# Supplementary material for: SARS-CoV-2 infected children form early immune memory responses dominated by nucleocapsid-specific CD8+ T cells and antibodies
Source: Front Immunol. 2022 Nov 2;13:1033364. doi: 10.3389/fimmu.2022.1033364 (PMC9667737; doi:10.3389/fimmu.2022.1033364)
Supplement: Supplementary file 1 [file DataSheet_1.pdf]

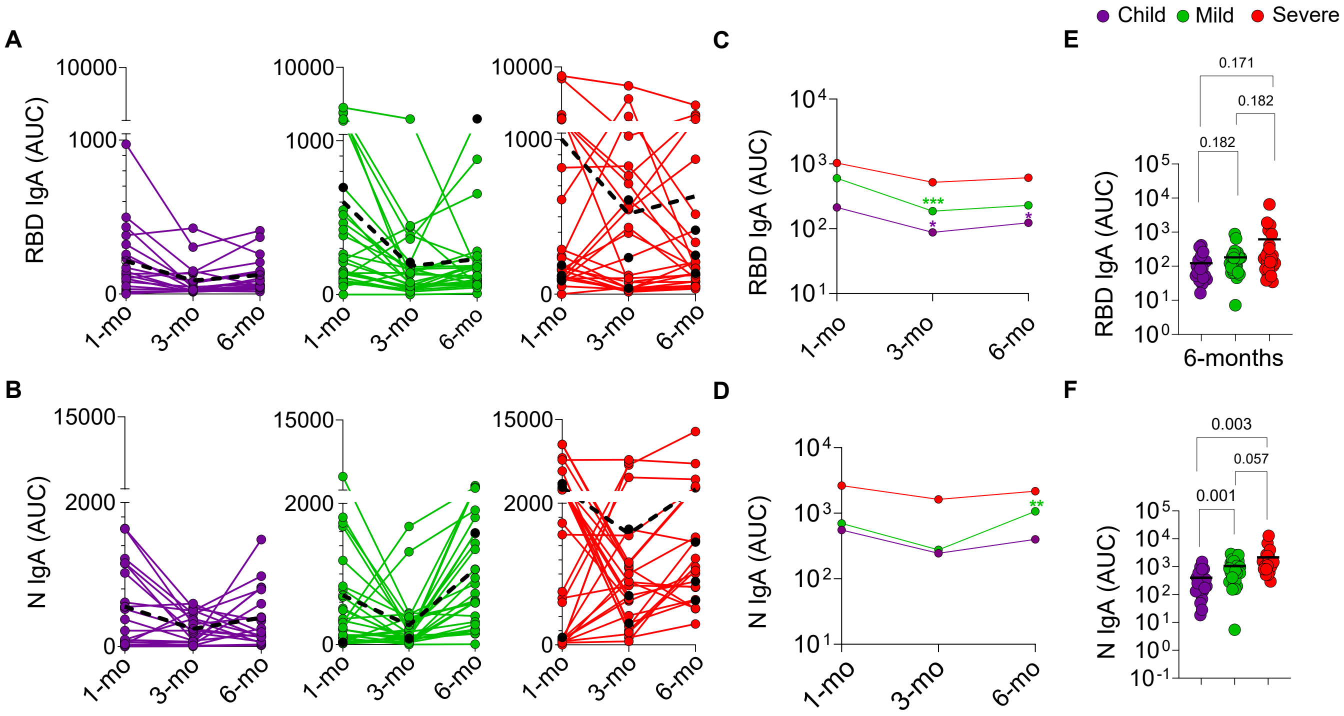

Figure S1. Analysis of IgA Antibodies. (A-B) Plasma IgA antibody binding to SARS-CoV-2 RBD and N protein, respectively, determined by enzyme-linked immunosorbent assay (ELISA), (n=70). Longitudinal analysis through connecting lines, color coded, represented as area under the curve (AUC). Dotted lines in black highlight median values. Individuals colored in black were vaccinated between the second and the third collection point (Mild with Astrazeneca and Severe with Sinovac). **(C-D)** median titers plotted over time, colored asterisk (*) indicate statistical difference with previous point. **(E-F)** comparison of individual AUC between groups 6-months after acute infection. Differences among groups were tested using Generalized Estimating Equations (GEE), while Sequential Sidák's adjustment was used in the pairwise multiple comparisons. P values are displayed over brackets, the horizontal bar representing the median.

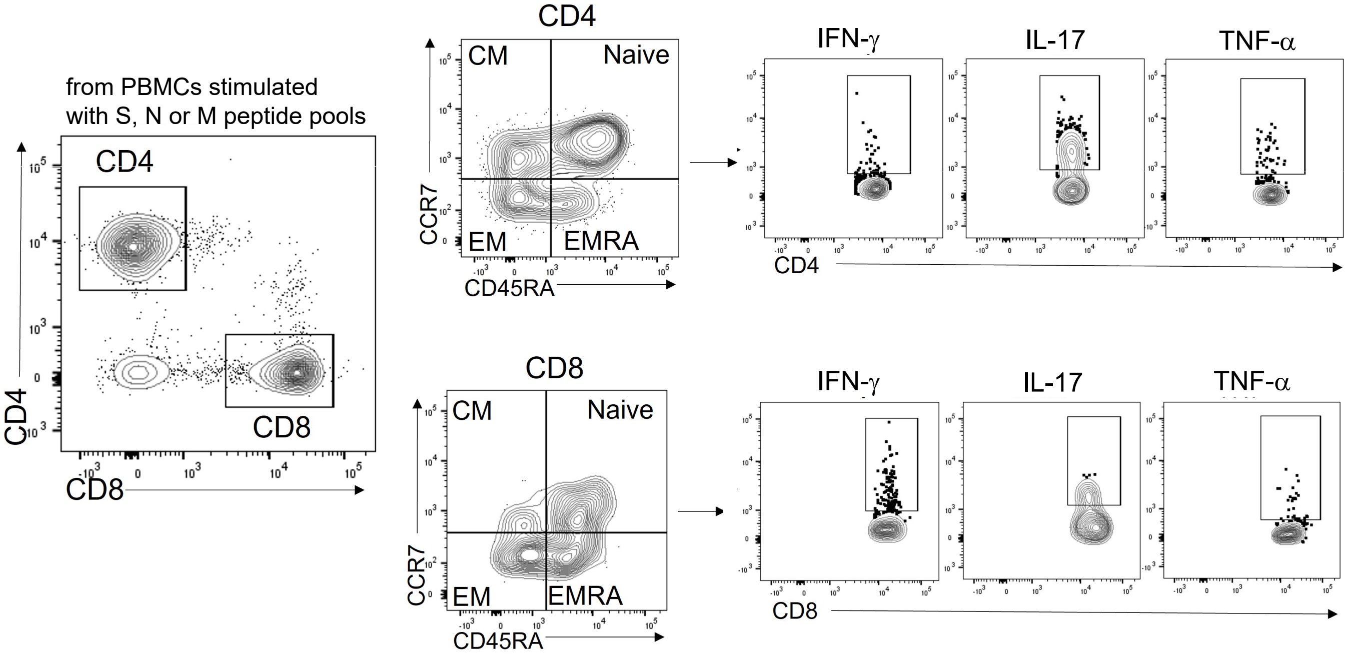

Fig. S2. Gating strategies for flow cytometry analysis of specific memory T cell in vitro responses. Analysis of memory CD4 and CD8 cells, through surface markers CCR7 and CD45RA, and functional intracellular markers IFN-γ, IL-17 and TNF-α.

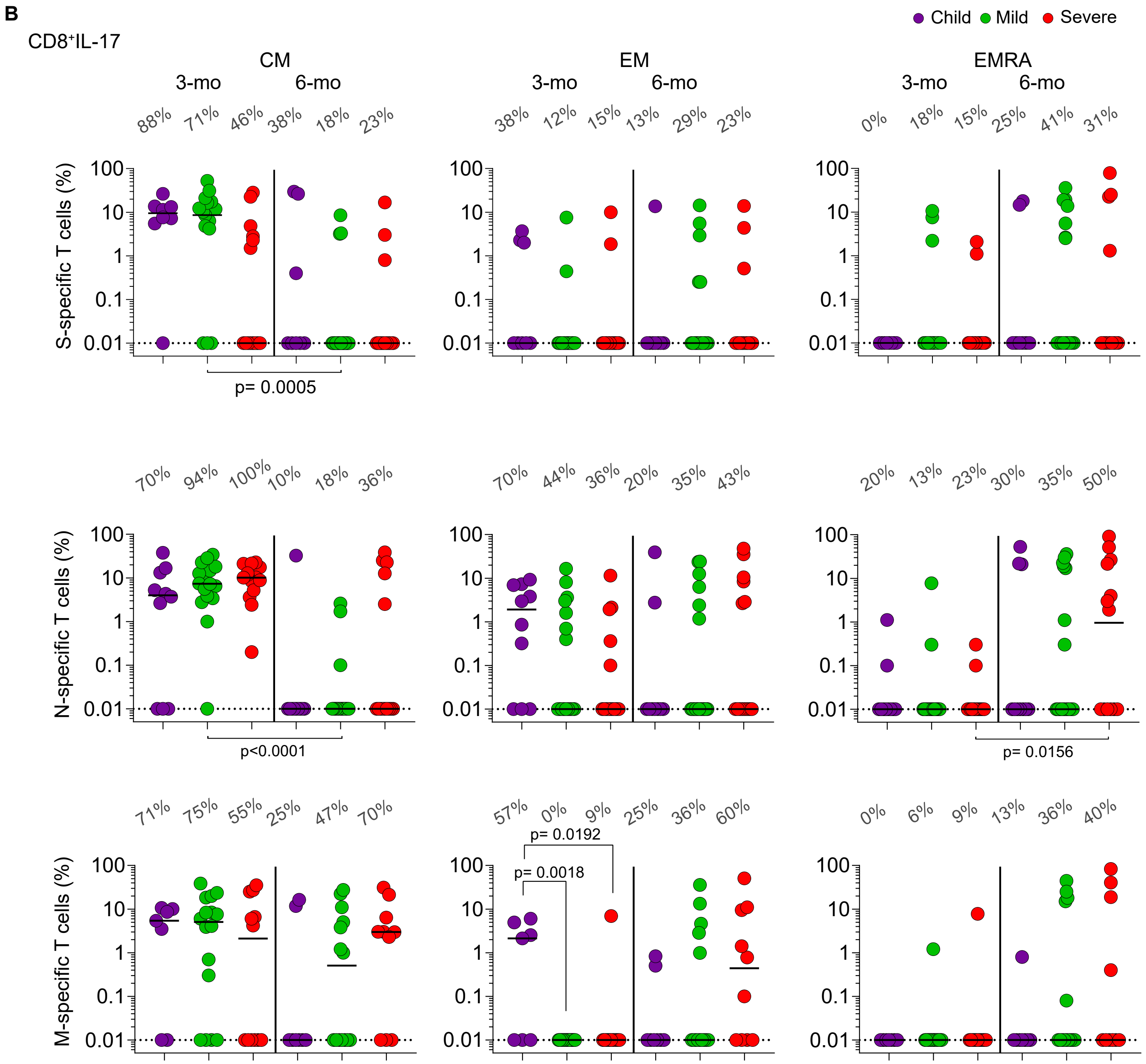

Figure S3. Longitudinal analysis of functional specific CD8 memory T cells. Individual frequencies of memory T cells collected at 3- and 6-months post-acute infection, responding to *in vitro* challenge with different SARS-CoV-2 peptide pools to proteins S, N or M. Considering CD8+INF-γ **(A)** and CD8+IL-17 **(B)** production after stimulation with pools. Differences between patients' groups at the same collection point were assessed by Kruskal-Wallis and Dunn's post hoc test; differences between collection points were assessed by Wilcoxon test.

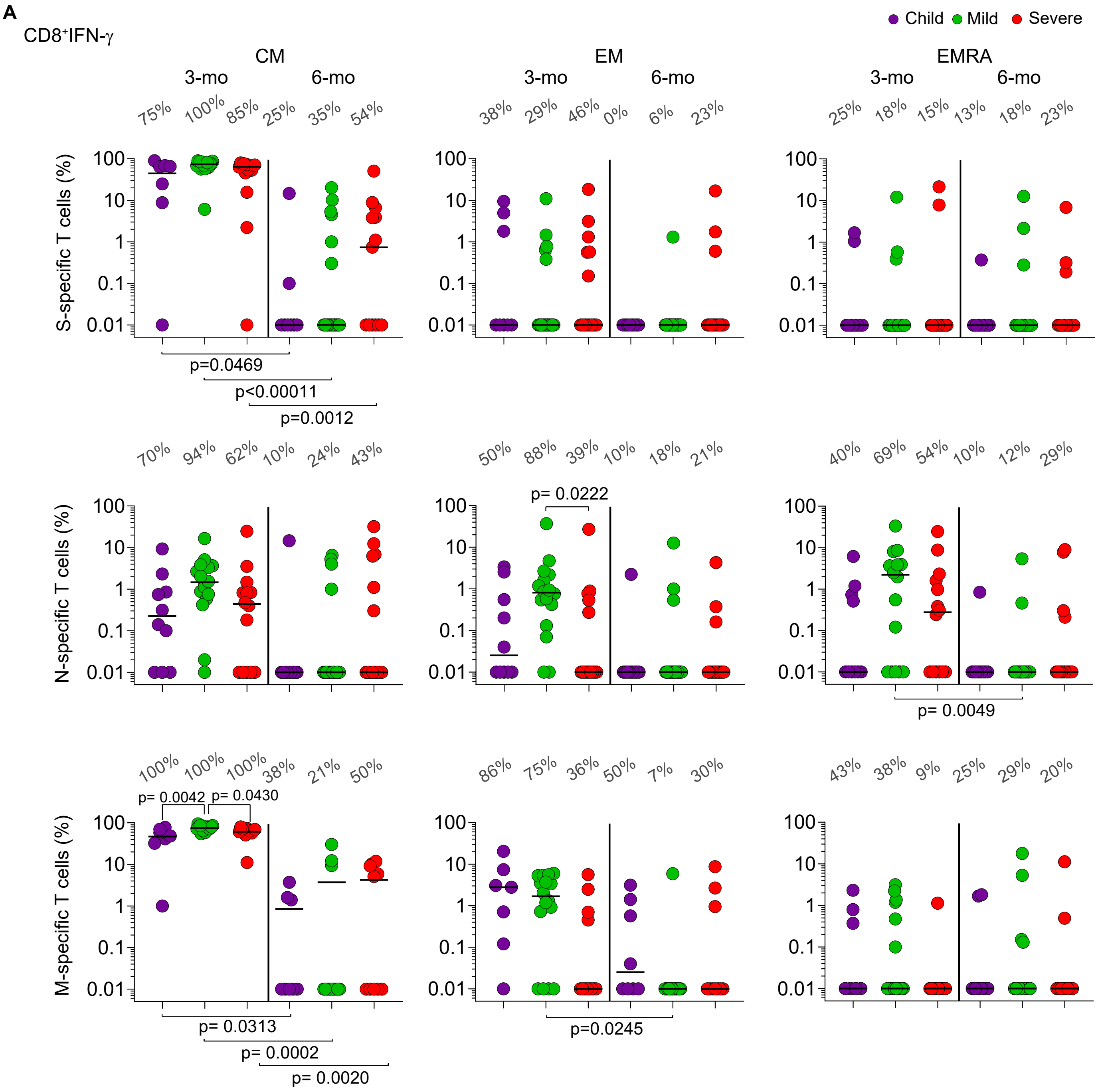
A
CD8⁺IFN-γ
Child Mild Severe
CM
EM
EMRA
3-mo
6-mo
S-specific T cells (%)
N-specific T cells (%)
M-specific T cells (%)
75% 100% 85% 25% 35% 54%
38% 29% 46% 0% 6% 23%
25% 18% 15% 13% 18% 23%
p=0.0469
p<0.00011
p=0.0012
70% 94% 62% 10% 24% 43%
50% 88% 39% 10% 18% 21%
40% 69% 54% 10% 12% 29%
p= 0.0222
p= 0.0049
100% 100% 100% 38% 21% 50%
86% 75% 36% 50% 7% 30%
43% 38% 9% 25% 29% 20%
p= 0.0042
p= 0.0430
p= 0.0313
p= 0.0002
p= 0.0020
p=0.0245

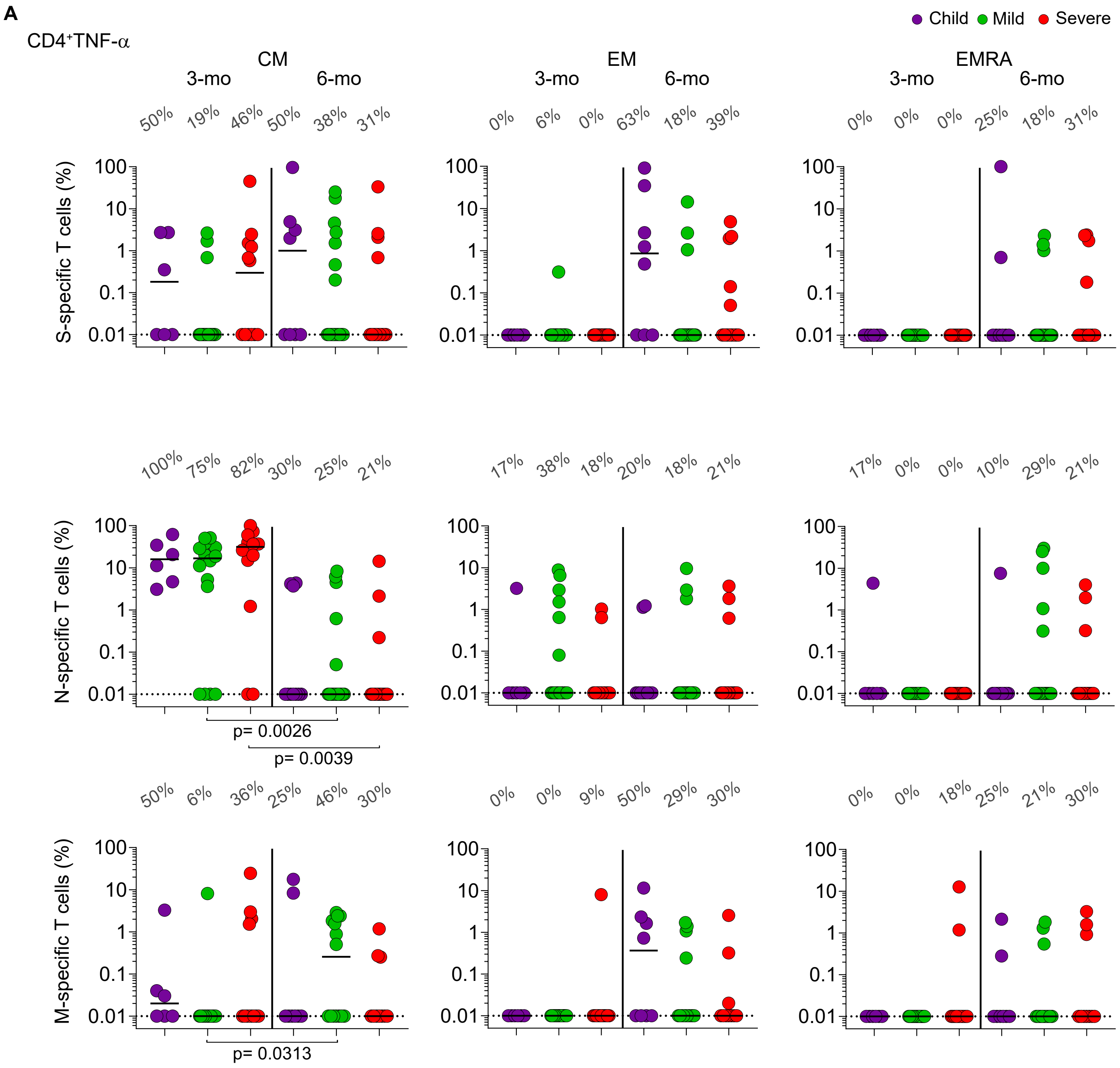
A
CD4+TNF-α
Child Mild Severe
CM
EM
EMRA
3-mo
6-mo
50% 19% 46% 50% 38% 31%
0% 6% 0% 63% 18% 39%
0% 0% 0% 25% 18% 31%
100% 75% 82% 30% 25% 21%
17% 38% 18% 20% 18% 21%
17% 0% 0% 10% 29% 21%
50% 6% 36% 25% 46% 30%
0% 0% 9% 50% 29% 30%
0% 0% 18% 25% 21% 30%
S-specific T cells (%)
N-specific T cells (%)
M-specific T cells (%)
100
10
1
0.1
0.01
p= 0.0026
p= 0.0039
p= 0.0313

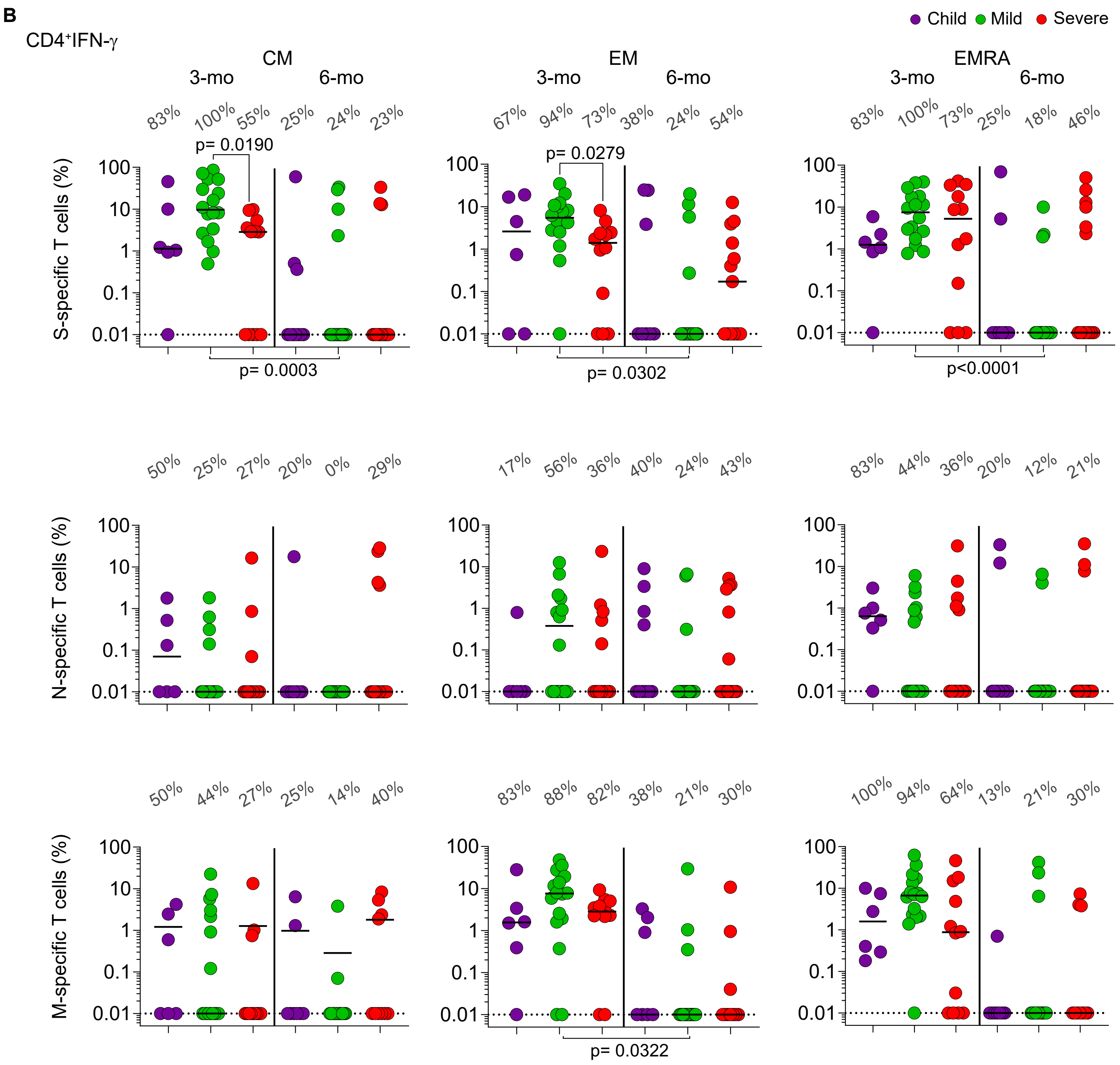

B
CD4+IFN-γ
Child
Mild
Severe
CM
EM
EMRA
3-mo
6-mo
83%
100%
55%
25%
24%
23%
p= 0.0190
p= 0.0003
67%
94%
73%
38%
24%
54%
p= 0.0279
p= 0.0302
83%
100%
73%
25%
18%
46%
p<0.0001
S-specific T cells (%)
50%
25%
27%
20%
0%
29%
17%
56%
36%
40%
24%
43%
83%
44%
36%
20%
12%
21%
N-specific T cells (%)
50%
44%
27%
25%
14%
40%
83%
88%
82%
38%
21%
30%
p= 0.0322
100%
94%
64%
13%
21%
30%
M-specific T cells (%)
100
10
1
0.1
0.01

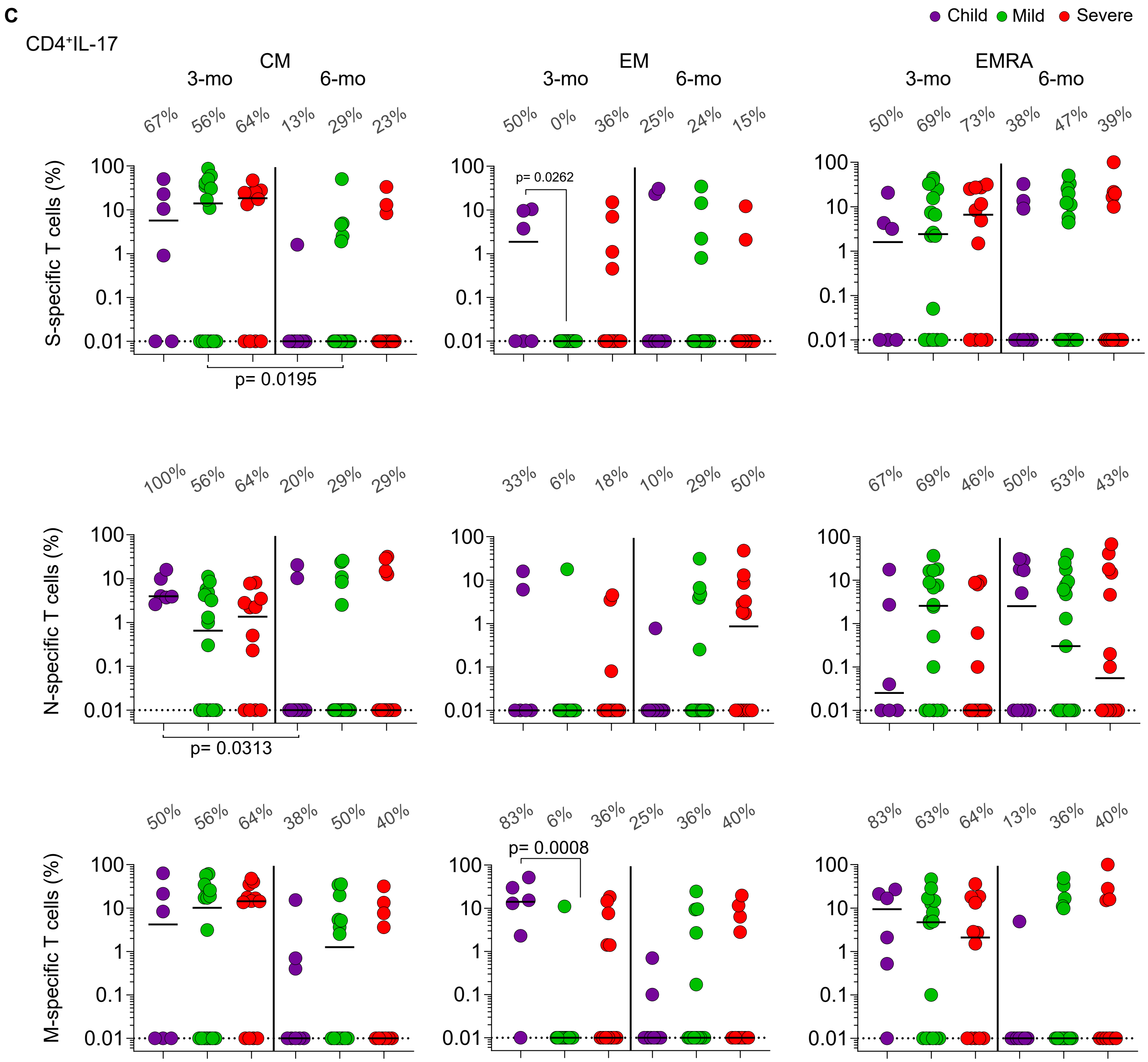

Figure S4. Longitudinal analysis of functional specific CD4 memory T cells. Individual frequencies of memory T cells collected at 3- and 6-months post-acute infection, responding to *in vitro* challenge with different SARS-CoV-2 peptide pools to proteins S, N or M. Considering CD4+TNF-α **(A)**, CD4+INF-γ **(B)** and CD4+IL-17 **(C)** production after stimulation with pools. Differences between patients' groups at the same collection point were assessed by Kruskal-Wallis and Dunn's post hoc test; differences between collection points were assessed by Wilcoxon test.

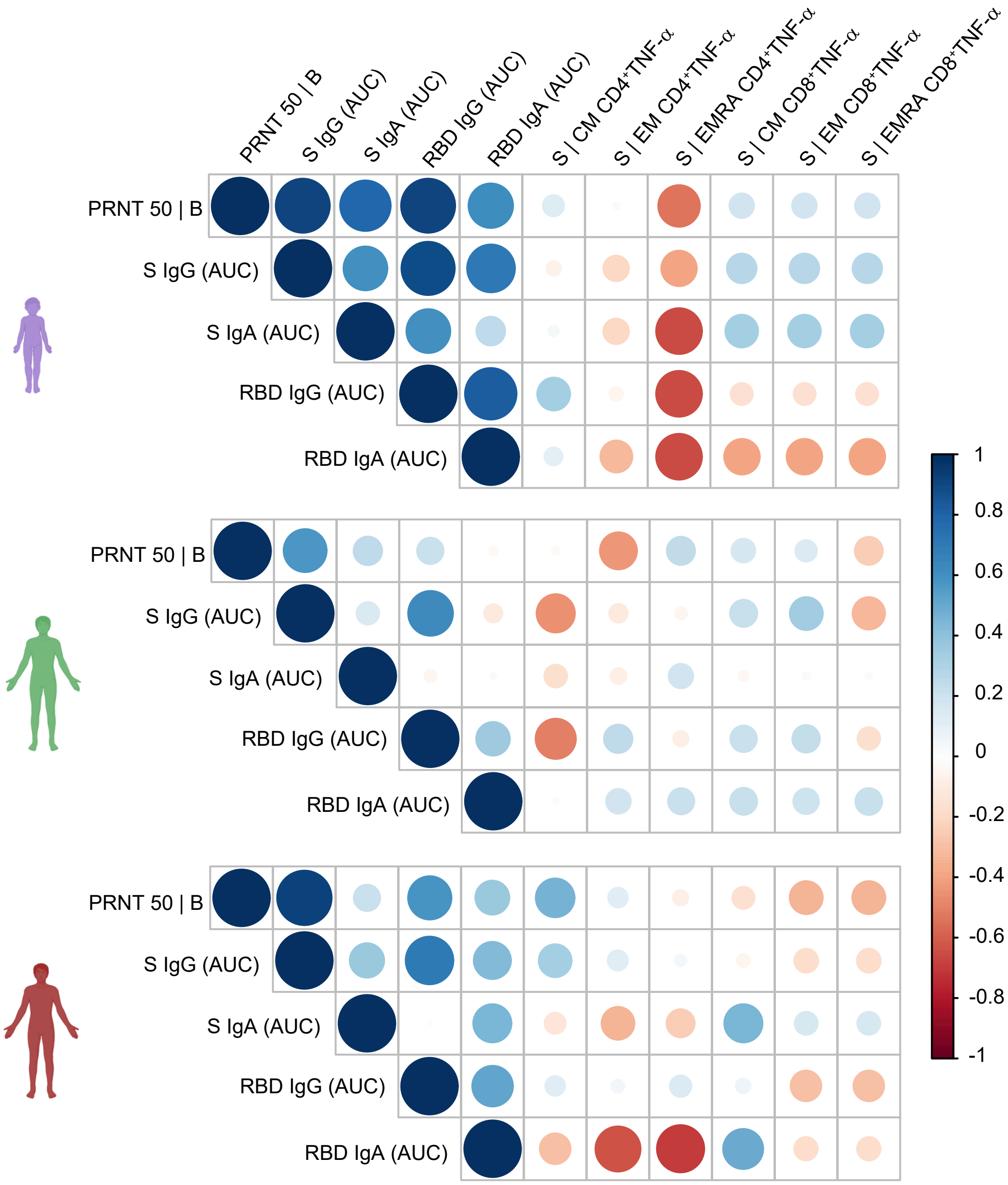

Figure S5. Spearman correlation analysis of antibody and S-protein specific TNF-α+ T cell responses, 6-months after acute infection. The matrix represents the correlation analysis of S-specific effector T cells responses (in percentages of CD4+ and CD8+ cells expressing cytokine TNF-α in response to the peptide pools), neutralizing antibodies to the B original variant, as well as antibody responses to the RBD protein (represented as values for the area under the curve - AUC). n children = 6, n mild = 16, n severe = 10.
