# Supplementary material for: SARS-CoV-2 infected children form early immune memory responses dominated by nucleocapsid-specific CD8+ T cells and antibodies
Source: Front Immunol. 2022 Nov 2;13:1033364. doi: 10.3389/fimmu.2022.1033364 (PMC9667737; doi:10.3389/fimmu.2022.1033364)
Supplement: Supplementary file 2 [file DataSheet_2.pdf]

Supplementary Note 1. COVIDa study group

Luciane Beatriz Kern, Thaís Raupp Azevedo, Maristênia Machado Araújo, Amanda Paz Santos, Shirlei Villanova Ribeiro, Fernando Rovedder Boita, Camila Dietrich, Fernanda Lutz Tolves, Jaina da Costa Pereira, Adriana Isabel Rohden, Thainá Dias Luft, Shirlei Villanova Ribeiro, Catia Moreira Guterres, Caroline Cabral Robinson, Débora Vacaro Fogazzi, Regis Goulart Rosa, Ana Paula dos Santos, Gisele Alcina Nader Bastos, Denise Arakaki-Sanchez, Maicon Falavigna, Patricia Bartholomay Oliveira e Francieli Fontana Sutile Tardetti Fantinato.
